# Chromosomal capture of beneficial genes drives plasmids toward ecological redundancy

**DOI:** 10.1093/ismejo/wraf091

**Published:** 2025-05-11

**Authors:** R Craig MacLean, Cédric Lood, Rachel M Wheatley

**Affiliations:** University of Oxford, Department of Biology, 11a Mansfield Rd., Oxford OX1 3SZ, United Kingdom; All Souls College, High Street, Oxford OX1 4AL, United Kingdom; University of Oxford, Department of Biology, 11a Mansfield Rd., Oxford OX1 3SZ, United Kingdom; School of Biological Sciences, Queen’s University Belfast, Belfast BT9 5DL, United Kingdom; Theoretical Sciences Visiting Program, Okinawa Institute of Science and Technology Graduate University, Okinawa 904-0495, Japan

**Keywords:** plasmid paradox, fitness, Tn-seq, bacterial evolution, *Rhizobium*

## Abstract

Plasmids are a ubiquitous feature of bacterial genomes, but the forces driving genes and phenotypes to become associated with plasmids are poorly understood. To address this problem, we compared the fitness effects of chromosomal and plasmid genes in the plant symbiont *Rhizobium leguminosarum*. The relative abundance of beneficial genes on plasmids was very low compared to the chromosome across niches that reflect key steps in plant colonization. Two lines of evidence support the hypothesis that this pattern emerges because evolutionary processes drive beneficial genes to move from plasmids to the bacterial chromosome. First, weakly beneficial genes that increased fitness in a single niche were evenly distributed between plasmids and the chromosome, whereas the chromosome was enriched for strongly beneficial genes that increased fitness across multiple niches. Second, beneficial genes were more prevalent on recently acquired plasmids compared to ancient plasmids. Our findings support a model in which bacterial lineages initially acquire plasmids due to the beneficial genes that they carry, but the movement of beneficial genes to the chromosome gradually erodes the ecological value of plasmids. These findings reconcile existing models of plasmids and highlight the challenge of understanding how plasmids can persist over the long term.

## Graphical Abstract

**Graphical Abstract:**
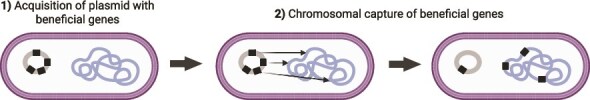

## Introduction

Bacterial genomes are made up of chromosomes and plasmids that replicate independently of the chromosome. Genes are continuously transferred between plasmids and chromosomes by transposons [1, 2], and uncovering the processes that drive genes and phenotypes to be associated with plasmids as opposed to bacterial chromosomes is a fundamental challenge in microbial ecology and evolution [1, 3–9].

The dominant view in microbiology is that plasmids play a key role in bacterial adaptation through the horizontal transfer of genes that are beneficial in defined ecological niches [10–14], such as genes associated with antibiotic resistance, pathogen virulence, or novel metabolic pathways [3, 9, 15–18]. However, classic evolutionary models that allow genes to move between plasmids and the chromosome predict that beneficial genes should become associated with chromosomes, as opposed to plasmids, questioning the role of plasmids in bacterial adaptation [4, 19]. It has been challenging to reconcile these two views of plasmids [7, 9, 12, 13, 20, 21] because the relative ecological and evolutionary importance of plasmid genes remains poorly understood beyond the paradigmatic examples highlighted above.

**Figure 1:**
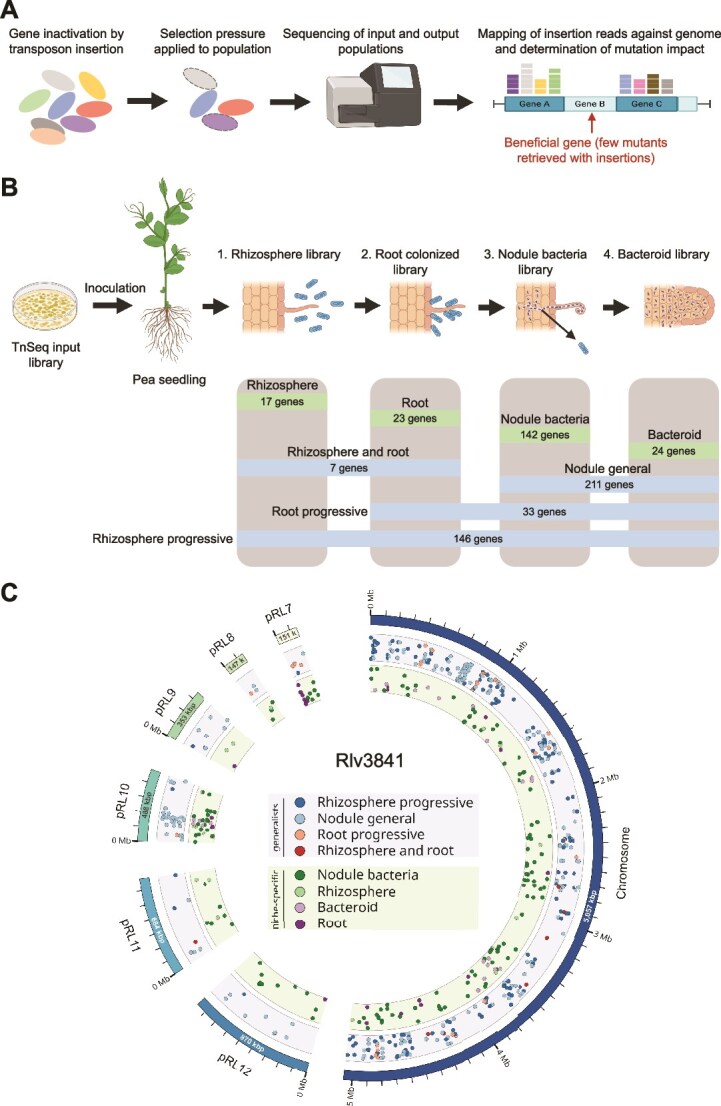
Testing for selection on genes by Tn-seq. (A) Schematic of a transposon insertion sequencing experiment. A mutant library is constructed using transposon insertion to inactivate genes on a genome-wide scale. When a selection pressure is applied to the population, mutants change in frequency in the population depending on the contribution of their mutated gene to fitness under that condition. The resulting populations are then sequenced and mapped back against the genome to determine the position and frequency of transposon insertion mutants across the genome relative to the input library that was used to start the experiment. Mutants of genes which are important for fitness will fall out of frequency of the population, allowing their identification as beneficial genes. (B) This study used source data from a previously published study in which an *R. Leguminosarum* mutant library was screened across multiple stages of symbiosis: Growth in the rhizosphere, root colonisation, nodule formation, and bacteroid formation [22]. The table indicates the number of genes that were identified to be beneficial across the corresponding symbiosis stages. Blue blocks indicate the number of genes beneficial in single niches, and green blocks indicate the number of genes beneficial in multiple niches. (C) The seven replicons of the *Rhizobium* genome are displayed on the outer circle of this circos visualization [48]. Genes that were beneficial in a single niche (green inner band) or across multiple niches (generalist genes; blue inner band) are marked for each replicon. Jitter was added along the y-axis (height) position of the circles to aid visualization of genes in close proximity.

Here we address this problem by systematically measuring the impact of plasmid and chromosomal genes on bacterial fitness in the plant symbiont *R. leguminosarum* using previously published data sets from a Tn-seq experiment [22]. Transposon insertions [23] were used to systematically mutagenize non-essential genes in the genome of a strain of *R. leguminosarum* carrying a chromosome and six plasmids [18]. Populations of pooled insertion mutants were then assayed by deep sequencing under conditions that recapitulate the ecology of *Rhizobium* [24], including growth in the rhizosphere, root colonization, nodulation, and bacteroid formation (Fig. 1A, B). The use of fitness assays under natural conditions is a key feature of this data set, given that plasmids are predicted to carry ecologically relevant genes whose effects may be missed in standard culture-based measures of bacterial fitness. This experiment uncovered 603 unique genes that were beneficial in either a single niche (specialist genes) or across multiple niches (generalist genes) (Fig. 1B and C ).

## Materials and methods

Our study used published data sets from a large-scale transposon insertion sequencing experiment that was used to identify genes in *R. leguminosarum* bv. *viciae* 3841 (Rlv3841) that underpin bacterial fitness during the establishment of symbiosis with the legume host pea (*Pisum sativum*) [22]*.* Genes that influence fitness were identified by sequencing pools of transposon mutants that were recovered from the rhizosphere, plant roots, nodules, and bacteroids and comparing the density of transposon mutants in the mutant pools with an initial input library that was used to inoculate the plants (Fig. 1). A hidden Markov model was applied to classify genes into one of four state classifications in each niche based on read counts. Genes were classified as essential (ES; no or very few insertions), growth defective (DE; low insertion read count), growth advantaged (AD; high insertion read counts), and neutral (NE; within the boundaries of a mean parameter of insertion read counts). This experiment was validated by testing the roles of 15 genes in follow-up experiments using independently constructed mutants. Only a single mutant did not recapitulate its predicted phenotype inferred from the sequencing of pooled populations of transposon mutants.

For our analysis, we downloaded the supplementary data (Dataset S01 and Tables S1–S8) from the source study [22]. The total number of genes assayed by Tn-seq in each replicon was calculated based on the number of genes where insertion mutants were detected in the input library. This was marginally less than the total number of genes on each replicon because some genes did not contain transposon insertions, suggesting that either insertions in the gene were lethal or that the transposon was unable to insert into the gene. As a final correction we excluded a small number of duplicate genes (*n* = 110) in the Rlv3841 genome from the analysis [25] due to the uncertainties in mapping transposon mutants to different copies of duplicated genes. We calculated the number of genes that were beneficial for each combination of replicon and niche by adding together the number of genes that were categorized as essential or growth defective in reference [22].

## Results

### Plasmids are depleted in beneficial genes

**Figure 2:**
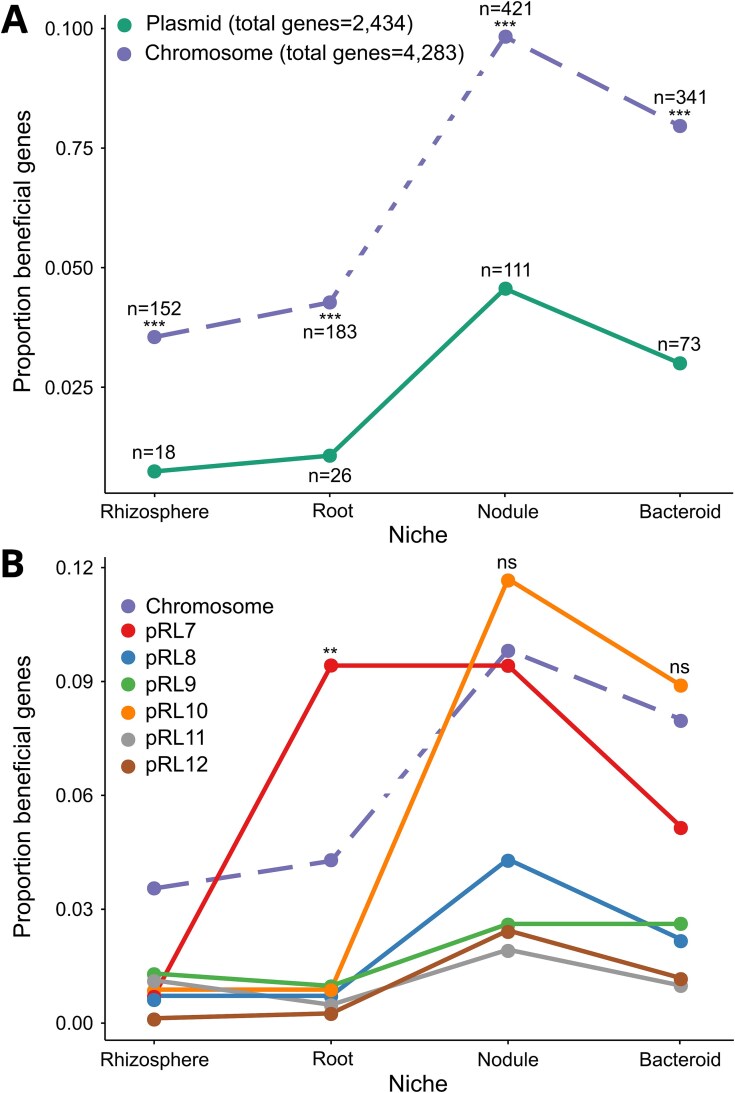
Plasmids are depleted in beneficial genes. Plots show comparisons of the prevalence of beneficial genes (i.e. beneficial genes/total genes) between the chromosome and all plasmids combined (A) and between individual replicons (B). We compared the proportions of beneficial genes on plasmids and the chromosome using a normal approximation to the binomial distribution. All comparisons between plasmids and the chromosome in A were statistically significant under a two-tailed null hypothesis with *P* < 1 × 10^−10^. In B we tested for an increased prevalence of beneficial genes on plasmids compared to the chromosome. Plasmid pRL7 was enriched in root adaptive genes compared to the chromosome under a one-tailed null hypothesis (*P* = .0017, ^**^).

To understand the benefits of plasmid and chromosomal genes, we calculated the fraction of genes that were beneficial in each niche. The proportion of plasmid genes with beneficial effects on fitness was low relative to the chromosome in all niches (Fig. 2A). This effect was strong, and beneficial genes were enriched on the chromosome compared to plasmids between 2.15 times (nodulation) and 4.80 times (rhizosphere). This simple result challenges the ecological importance of plasmids.

A limitation of summing beneficial genes across plasmids is that it treats plasmid genes as a collective. If plasmids are key drivers of niche adaptation, then individual plasmids might be associated with genes involved in specialization on distinct niches. Consistent with this idea, we found two clear examples of niche-associated plasmids. Plasmid pRL10 carries genes that play important roles in the establishment of symbiotic interactions with legumes, including nitrogen fixation [18, 22]. As expected, this plasmid was associated with genes that were beneficial during nodulation and bacteroid formation. Second, plasmid pRL7 was associated with genes that were beneficial across all niches associated with plants, including root colonization. Although these examples highlight the association between plasmids and niches, plasmids were not enriched in niche-adaptive genes compared to the chromosome, except for a single case of genes involved in root colonisation on plasmid pRL7.

### Plasmids are associated with niche specialist genes

**Figure 3:**
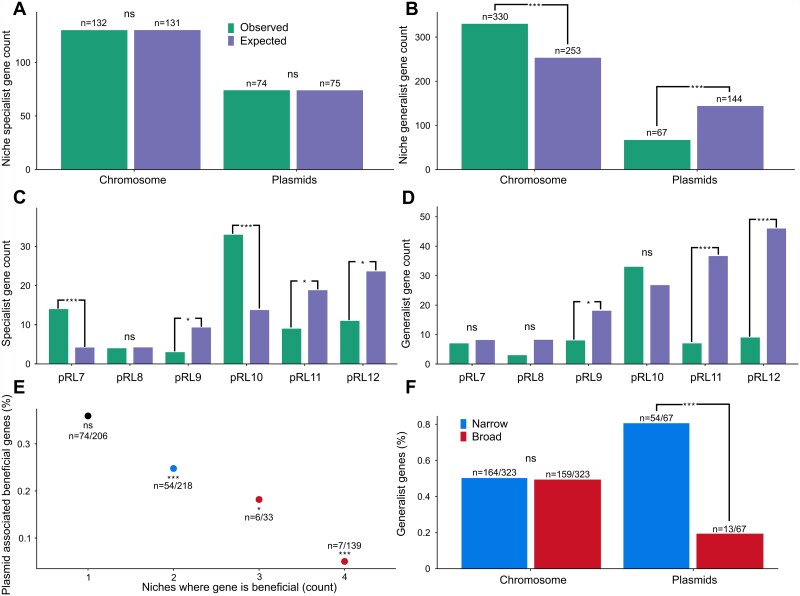
Plasmids are associated with niche specialist beneficial genes. Bar charts show the observed and expected number of specialist (A,C) and generalist (B,D) genes across the genome. Panels A and B show a comparison of all plasmid genes with the chromosome, and panels C and D show individual plasmid replicons, with observed gene counts shown in green and expected gene counts shown in dark blue. Expected gene numbers were calculated based on the number of genes on each replicon under the null hypothesis that the prevalence of beneficial genes is equal for all replicons. We tested for beneficial gene enrichment using two-tailed binomial tests comparing all plasmids and the chromosome (A,B) or individual plasmid replicons (C,D). Statistical tests for individual replicons were corrected for multiple testing using the Bonferroni correction. Panel E shows the proportion of beneficial genes associated with plasmids as a function of the number of niches where the gene was beneficial. The number of plasmid-associated beneficial genes is shown and we tested the null hypothesis that beneficial genes are evenly distributed across the genome using two-tailed binomial tests. Panel F shows the proportion of generalist genes that increased fitness in a narrow range (2 niches) or a broad range (3 or 4 niches) of niches for plasmids and the chromosome. We tested for a difference in the proportion of narrow and broad range generalist genes using a normal approximation to the binomial distribution (n.s, no significant enrichment, ^*^, *P* < .05, ^**^, *P* < .01, ^***^, *P* < .001).

If evolutionary processes drive beneficial genes to become localized to the chromosome, genes that are under strong selection should be more likely to be associated with the chromosome compared to genes that are under weak selection [19]. To test this prediction, we compared the distribution of genes that were beneficial in a single niche (specialist genes) with those that were beneficial across multiple niches (generalist genes). The underlying assumption of this test is that genes that are beneficial in a single niche are under weak selection compared to genes that are beneficial across multiple niches when selection is considered across the entire life cycle of *Rhizobium*. As a collective, plasmids were not enriched in specialist genes compared to the chromosome (Fig. 3A). However, pRL10 and pRL7 were enriched in specialist genes, reflecting the roles that these plasmids play in interactions between *Rhizobium* and plants. The overall lack of specialist genes on plasmids was driven by the low abundance of niche specialist genes on the remaining plasmids, particularly pRL11 and pRL12. In contrast, generalist genes that were beneficial across multiple niches were strongly associated with the chromosome (Fig. 3B). None of the plasmid replicons were enriched in generalist genes, and the depletion of generalist genes was particularly clear for plasmids pRL9, pRL11, and pRL12.

To further test the hypothesis that genes under strong selection become associated with chromosomes, we treated the number of niches where genes were beneficial as an ordinal variable (i.e. 1–4 niches) as opposed to a binary variable (i.e. specialist or generalist). As expected, plasmids were depleted in genes that were beneficial across multiple niches compared to the chromosome (Fig. 3E). An alternative way to visualize this result is to compare the prevalence of narrow-range generalist genes that were beneficial in 2 niches with broad-range generalist genes that were beneficial in 3 or 4 niches (Fig. 3F). Almost all generalist genes carried by plasmids were narrow-range, whereas broad- and narrow-range generalist genes were equally represented on the chromosome.

### Plasmids lose beneficial genes over time

**Figure 4:**
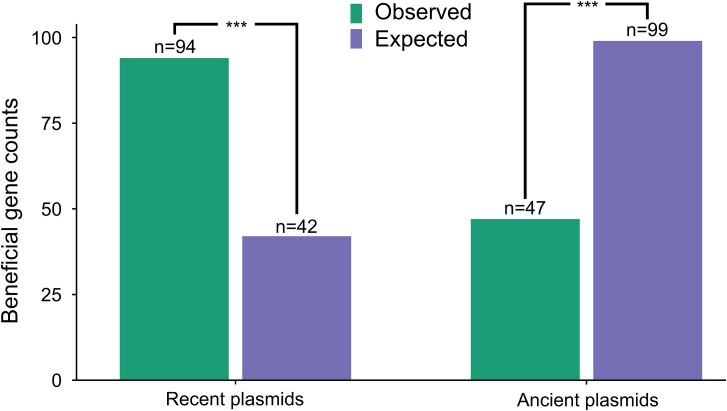
Ancient plasmids are depleted in beneficial genes. Bar charts show the expected and observed number of beneficial genes for recently acquired (pRL7, pRL8, pRL10) and ancient (pRL9, pRL11, pRL12) plasmids. Expected gene numbers were calculated based on the number of genes on each replicon under the null hypothesis that the prevalence of beneficial genes is equal across plasmids. We tested for significant deviations from expected gene counts using a two-tailed binomial test, and both *P* values were highly significant (*P* < 6 × 10^−4^).

If selection favors the movement of beneficial genes from plasmids to the chromosome, then recently acquired plasmids should be rich in beneficial genes compared to ancient plasmids. Plasmids pRL9, pRL11, and pRL12 lack motility systems and have a nucleotide composition that matches the chromosome, suggesting that they were acquired by *Rhizobium* in the distant past [18]. The remaining plasmids (pRL7, pRL8, pRL10) have divergent nucleotide composition from the chromosome and plasmid mobilization systems (pRL7 and pRL8), implying that they have been more recently acquired [18]. All these plasmids have *repABC* operons that control plasmid partitioning and replication [18, 26], implying that the number of housekeeping genes is very low and comparable between plasmids. To test this hypothesis, we compared the prevalence of all beneficial genes between recently acquired and ancient plasmids (Fig. 4). We did not distinguish between specialist and generalist genes in this analysis, as plasmids carried few generalist genes that were typically beneficial in only 2 niches (Fig. 3E,F). Beneficial genes were over-represented on recently acquired plasmids compared to ancient plasmids, suggesting that plasmids become gradually depleted in beneficial genes over time.

## Discussion

Plasmids are a ubiquitous component of bacterial genomes, but their role in adaptation remains unclear. Classic evolutionary models allowing movement of genes between chromosomes and plasmids predict that beneficial genes will eventually become localized to the chromosome [4]. Consistent with this model, we found that plasmids were depleted in beneficial genes compared to the chromosome (Fig. 2A), because the chromosome was strongly enriched in genes that were beneficial across multiple ecological niches (Fig. 3). If the chromosome effectively captures beneficial genes, then we would expect plasmids to undergo a process of gradual ecological decay due to the loss of beneficial genes. Consistent with this idea, we found that ancient plasmids were depleted in beneficial genes compared to recently acquired plasmids (Fig. 4). These findings support a model in which bacterial lineages initially acquire plasmids due to the beneficial genes that they carry, but the movement of beneficial genes to the chromosome gradually erodes the ecological value of plasmids, emphasizing the challenge of understanding how plasmids can persist over the long term [6, 7, 27–31].

The paradigm that plasmids play a key role in adaptation by providing bacteria with genes that are beneficial in specific ecological niches is deeply ingrained in microbiology [10–14]. As expected from this paradigm, we found that plasmids were associated with genes that increased fitness in specific ecological niches (Fig. 2B). One of the key insights from our study is that this association arises because evolution drives strongly beneficial genes, such as those that increase fitness across multiple niches, to become localized to the chromosome, leaving plasmids associated with niche specialist genes (Fig. 3). We argue that this link between plasmid degeneration and niche specialization reconciles the adaptationist view of plasmids that has emerged from empirical studies with evolutionary models that predict the degeneration of plasmids.

One limitation of our study is that we have not provided direct evidence of the transfer of beneficial genes from *Rhizobium* plasmids to the chromosome. Experimental studies have shown that transposons can rapidly move advantageous genes between plasmids and chromosomes [1, 2]. Some antibiotic resistance genes have moved from plasmids to the chromosomes of pathogenic bacteria [32–35], demonstrating that plasmid-to-chromosome transfer of beneficial genes occurs outside of the lab. However, the co-occurrence of homologous genes on plasmids and chromosomes in the same genome is rare across bacteria [36], including *R. leguminosarum* [25]. The lack of gene sharing between plasmids and chromosomes suggests that the transfer of beneficial genes from plasmids to chromosomes tends to be followed by the loss of redundant copies of selectively advantageous genes from plasmids. We speculate that the loss of plasmid copies of duplicate genes typically occurs at a slow rate but in some cases gene duplication is likely to be followed by rapid gene loss, such as when functional incompatibilities exist between plasmid and chromosomal copies of beneficial genes [37]. The inherent instability of duplicate genes makes it very difficult to find direct evidence of plasmid-to-chromosome gene transfer, and this is an important challenge for future research.

If selection favors the movement of beneficial genes from plasmids to chromosomes, why do plasmids carry beneficial genes? The ability of plasmids to transfer between bacterial strains and species provides plasmids with a unique opportunity to acquire novel beneficial genes [1]. In this case, the lack of beneficial genes on *Rhizobium* plasmids may in part be driven by the low mobility of these plasmids, which could restrict their ability to acquire novel beneficial genes from alternative bacterial hosts. Carrying beneficial genes on multi-copy plasmids can also be advantageous under some scenarios, such as when selection favors high levels of gene expression [2, 38] or heterogeneous gene expression between cells [39]. Multi-copy plasmids also allow genes to evolve in response to novel selective pressures at an accelerated rate [38], suggesting that it may be beneficial for genes under recurrent selection to be retained on plasmids. Finally, the potential for beneficial genes to move from plasmids to the chromosome may be restricted. Composite transposons are probably the most important vehicle for the movement of genes from plasmids to chromosomes, and the time needed for the formation of novel composite transposons [40] may represent an important constraint on the mobilization of genes from plasmids to chromosomes.

Many of the most important forms of antibiotic resistance have been driven by the dissemination of plasmids carrying antibiotic resistance genes [15, 41, 42]. Our findings predict that the strong selective pressures caused by the continued large-scale use of antibiotics will drive the integration of resistance genes into bacterial chromosomes [32–35]. This outcome may have complex impacts on antimicrobial resistance. One possible outcome is that the chromosomal integration of resistance genes might stabilize resistance in pathogen lineages, which can lose resistance plasmids due to the costs they impose [28, 43–45]. Alternatively, reducing the mobility of resistance genes could reduce the stability of resistance genes at the scale of bacterial communities by limiting the ability of resistance genes to transmit between bacteria [6, 46, 47].

## Supplementary Material

Supplementary_data_1_beneficial_genes_wraf091

Supplementary_file_summary_data_for_figures_wraf091

## Data Availability

All data analyzed in this study are included in this article and the supplementary material.

## Data and materials availability

This study used publicly available datasets downloaded from Wheatley *et al.* [22] (Dataset S01 and Tables S1–S8). Data sets used in this analysis are given in Supplementary Data File S1, and raw data for figures are in Supplementary Data File S2.
